# A Digital Tool for Assessing the Distinct Effects of Depression, Anxiety, and Attention-Deficit/Hyperactivity Disorder (ADHD) on Children’s Emotional Cognitive Bias: Cross-Sectional Study

**DOI:** 10.2196/86286

**Published:** 2026-02-25

**Authors:** Sang-Eon Park, Jisu Chung, Sang Ah Lee

**Affiliations:** 1 Department of Brain and Cognitive Sciences Seoul National University Seoul Republic of Korea; 2 Brain Imaging Center Seoul National University Seoul Republic of Korea; 3 Institute for Data Innovation in Science Seoul National University Seoul Republic of Korea

**Keywords:** ADHD, attention, attention-deficit/hyperactivity disorder, eHealth, emotion, mental health

## Abstract

**Background:**

Emotional wellness and healthy neurocognitive development are crucial from early childhood. An imbalance in attentional and emotional regulation system is associated with an increased risk of depression, anxiety, and attention-deficit/hyperactivity disorder (ADHD). Early assessment of these risks is essential, but it is difficult to conduct cognitive tests that are both child-friendly and able to dissociate different behavioral biases.

**Objective:**

This study aimed to develop a digital app–based tool designed for young children to objectively assess cognition-affect interactions and examine the association with standardized scales for anxiety, depression, and ADHD.

**Methods:**

In this cross-sectional study, 78 healthy children (36 female) aged 4-10 (mean 7.2, SD 1.4) years with no history of mental illness were recruited from the local community center and children’s museum. Emotional regulation and attentional control were assessed using an animated emotional Flanker task, emotional Stroop task, and emotional Go/No-Go task on a touchscreen computer. Children’s current mental health was measured using self-reported depression and anxiety states through the Center for Epidemiological Studies Depression Scale for Children (CES-DC) and State-Trait Anxiety Inventory for Children (STAI-CH), while the ADHD risk was assessed using the Korean ADHD Rating Scale (K-ARS) for parents. Principal component analysis was applied to behavioral measures across the tasks to group them by similarities and extract 3 abstract scores (“E-scores”) representing different aspects of cognitive function (attention, selective inhibition, and emotional sensitivity). Associations between E-scores and mental health or ADHD risk were then tested.

**Results:**

There was a significant improvement in general attention across development (Pearson correlation between E-score 1 and age: *r*=–0.75; *P*<.001; 2-sided α=.05) but not emotion-attention interactions. Performance was also correlated with mental health scales. First, children with higher depression symptoms (ie, higher CES-DC) were slower in their responses in general (ie, higher E-score 1; Pearson correlation after controlling for age: *r*=0.29; *P*=.04). Second, both anxious and depressed (ie, higher STAI-CH and CES-DC) children demonstrated reduced attention selectively to the emotional stimuli as indicated by elongated RT and lower accuracy (ie, higher E-score 2; anxiety: *r*=0.34; *P*=.02; depression: *r*=0.51; *P*<.001). Lastly, children with higher ADHD scales (ie, higher Korean ADHD Rating Scale [K-ARS]) showed lower accuracy across the three tasks, particularly for emotional stimuli (ie, lower E-score 3; *r*=–0.32; *P*=.03).

**Conclusions:**

By combining well-established emotional cognitive tasks with our dimensionality reduction techniques, we extracted individual affective-cognitive characteristics from diverse but noisy behavioral patterns and identified their association with mental health and ADHD-related symptoms in children. These results demonstrate the scientific validity, versatility, and translational potential of our gamified digital assessment tool for monitoring young children’s affective and cognitive health in daily life. Future longitudinal studies in children with formal clinical diagnoses will further strengthen the generalizability of these findings.

## Introduction

Early childhood is a crucial phase of cognitive and emotional development that sets the stage for children’s well-being throughout life. Disruptions in the healthy processing of emotional stimuli not only raise the risk of mental disorders such as depression, anxiety, and attention-deficit/hyperactivity disorder (ADHD), but also make it challenging for children to deploy and regulate attentional control, causing difficulties in children’s learning and school performance [1]. However, because these symptoms often begin at a young age [2], early monitoring and objective identification of individual vulnerability have been challenging based on traditional parent reports and clinical interviews.

Understanding how emotional processing in the brain develops during childhood provides insight into how such interactions between emotional and cognitive function are related to mental health. During childhood, the brain first undergoes subcortical development in emotion-processing regions such as the amygdala [3] and reward-processing regions such as the ventral striatum [4]. Across maturation, these regions form stronger network connectivity to the prefrontal cortex (PFC) [5], which develops gradually for modulating arousal and attention [5,6], ensuring that emotional stimuli are assessed and managed in a context-appropriate manner. Due to the immature regulatory function of the PFC, younger children rely more on subcortical function. This developmental imbalance causes them to exhibit greater sensitivity to emotional stimuli [7,8], which can be detrimental to both emotional wellness and cognitive performance from early childhood [9].

Based on findings from developmental cognitive neuroscience, the effects of depression, anxiety, and ADHD on cognitive abilities such as emotion recognition, attention, and inhibitory control may be dissociable despite their high comorbidity [10]. For instance, while depression is mainly characterized by feelings of sadness, low mood, and loss of interest in their usual activities [11], it is also associated with slower cognitive processing speed [12] and difficulty disengaging from negative emotional information [13]. Anxiety, on the other hand, is more strongly associated with heightened reactivity to potential threat [14,15], leading to an attentional bias toward emotional stimuli and an impairment in one’s ability to focus on nonthreatening, neutral stimuli [16]. Finally, although ADHD is marked by deficits in cognitive control, impulse regulation, and hyperactivity in daily life patterns [17], children with ADHD tend to be particularly more impulsive in response to rewarding or positively valenced stimuli [18,19].

Serious games can serve as digital monitoring tools that can be applied in daily life [20] and are particularly effective for young children who may be less receptive to repeated administrations of self-report surveys and cognitive tests. Recent studies have demonstrated that digital tools can be developed by leveraging associations between cognitive profiles and mental health or ADHD symptoms [21]. For example, one study assessed ADHD risk in children aged 4-8 years by examining cognitive functions such as reward processing using computer-based games [22]. Other studies have measured attention and working memory to examine their associations with depression [23] and anxiety [24] symptoms in adults. In addition, some studies did not directly evaluate the association between performance of digital cognitive tasks and mental health scales but instead assessed related cognitive domains, such as executive functions [25] and cognitive control [26]. However, most prior digital task–based studies have focused on single symptom domains, without accounting for the high comorbidity among ADHD, depression, and anxiety, which may not only necessitate separate tests for each but also limit the interpretability of assessments.

Unfortunately, despite the overwhelming evidence that mental health is simultaneously influenced by emotional sensitivity and cognitive processing [27-30], there is still a lack of digital tasks that capture such interactions within the scope of a game-like application that can be widely used by the public. More importantly, prior studies showed critical limitations in distinguishing the impacts of depression, anxiety, and ADHD simultaneously, using a single versatile assessment tool, despite the differences in the neurocognitive mechanisms that underlie their specific symptomatology [31]. The duration and user-friendliness of such tasks are especially critical for testing young children, as multiple lengthy traditional laboratory-based tests often fail to sustain children’s interest or use excessively violent or provocative emotional stimuli that are inappropriate for young children [32].

Given the above limitations, we set out to develop and validate a novel digital assessment tool that can characterize the relationships between children’s mental health or ADHD survey scores and emotional cognitive performance, an integration that has been largely absent from previous studies. Based on our previous study on a diverse range of both healthy participants and depressed adults [33], we designed a gamified task that is engaging and suitable for young children, which can be administered easily in about 30 minutes. A unique and novel aspect of our approach was to calculate abstract componential measures extracted from a range of behavioral patterns, which allowed us to identify individual cognitive-affective characteristics that were differentially associated with anxiety, depression, and ADHD symptoms. Unlike prior digital task–based tools that focus on isolated symptom domains, our framework enables simultaneous assessment of mental health and ADHD symptoms within a single test.

We developed modified versions of the emotional Flanker task (eFlanker), emotional Go/No-Go task (eGoNoGo), and emotional Stroop task (eStroop) using animated faces on a touchscreen interface. The eFlanker measured children’s attentional control in quickly assessing the perceptual stimuli and responding to the target stimulus without being influenced by nearby distractors. The eGoNoGo measured children’s ability to remember and select particular combinations of emotional and perceptual features, while asserting cognitive control to suppress responses to nontarget lures. Additionally, we developed a novel eStroop, requiring simple responses to the target features while inhibiting the irrelevant information. These 3 tasks demonstrated their validity as a psychometric test for children (aged 4-6 years) in previous studies [34-36]. Mental health and ADHD symptoms were assessed using standardized surveys that are widely used in both research and clinical settings.

To ensure the task was usable and relevant for the general public, we assessed children who may be at elevated risk for cognitive-affective difficulties but had not yet been diagnosed with a mental disorder. Recruitment in a community-based context allowed us to obtain a broad and representative dataset spanning healthy to at-risk individuals while minimizing explicit selection biases. We chose a target age range of 4-10 years, which corresponds to early childhood, a developmental period marked by ongoing maturation of prefrontal cortical systems supporting emotional regulation and cognitive control. This period is also when individual affective-cognitive characteristics related to anxiety, depression, and ADHD begin to emerge [37-39]. Given that adolescence is associated with distinct neurobiological mechanisms in processes such as risk-taking and emotion regulation [40], we limited recruitment to children younger than 10 years of age, in accordance with the definition of adolescence provided by the World Health Organization [41].

## Methods

### Participants

Children aged between 4 and 10 years without any history of mental illness were recruited from a local community center and a local children’s museum (Gyeonggi Children’s Museum; N=78; 36 female, mean age 7.2, SD 1.4 years). A convenience sampling approach in a public, community-based context was used to recruit children from diverse environments while minimizing explicit selection biases. Data were collected over a 6-month period (August 2023 to February 2024). This schedule was chosen to avoid the annual grade transition in March within the Korean education system, which can represent a substantial environmental change for children. Children who could not use the touchscreen were excluded from the study.

### Sample Size Determination

To determine the required sample size for this study, we referred to previous studies reporting associations between attention-related behavioral features and mental health or ADHD scales. In our previous work [33], emotion-related slowing of reaction time (RT) in the Flanker task (relative to the neutral condition) showed Pearson correlations of *r*=0.342 with depression symptoms and *r*=0.359 with anxiety symptoms. A power analysis showed that the minimum sample sizes required to achieve 80% statistical power (*β*=.2) with a 95% confidence level for these correlation coefficients were 64 and 59, respectively. In addition, we referred to another study reporting an association between error rate in the Flanker task and ADHD rating scale scores (Pearson correlation *r*=0.52) [42], for which the minimum required sample size under the same criteria was 27. To account for potential participant loss and missing data that could reduce statistical power, the target sample size was increased by 20%, resulting in a final sample size of 78 participants.

### Ethical Considerations

This study was conducted in accordance with the Declaration of Helsinki, and all experimental procedures were approved by the institutional review board for human research of Seoul National University (2501/003-021 and 2407/001-014). Written informed consent, which included all general elements required for research involving children, such as the purpose of the study, experimental procedures, potential benefits and discomforts, confidentiality, and the right to withdraw, was obtained from the parent or legal guardian. All study data were deidentified immediately after collection. All participants were compensated with 20,000 KRW (US $14) for 30 minutes of participation.

### Emotional Cognitive Tasks

**Figure 1:**
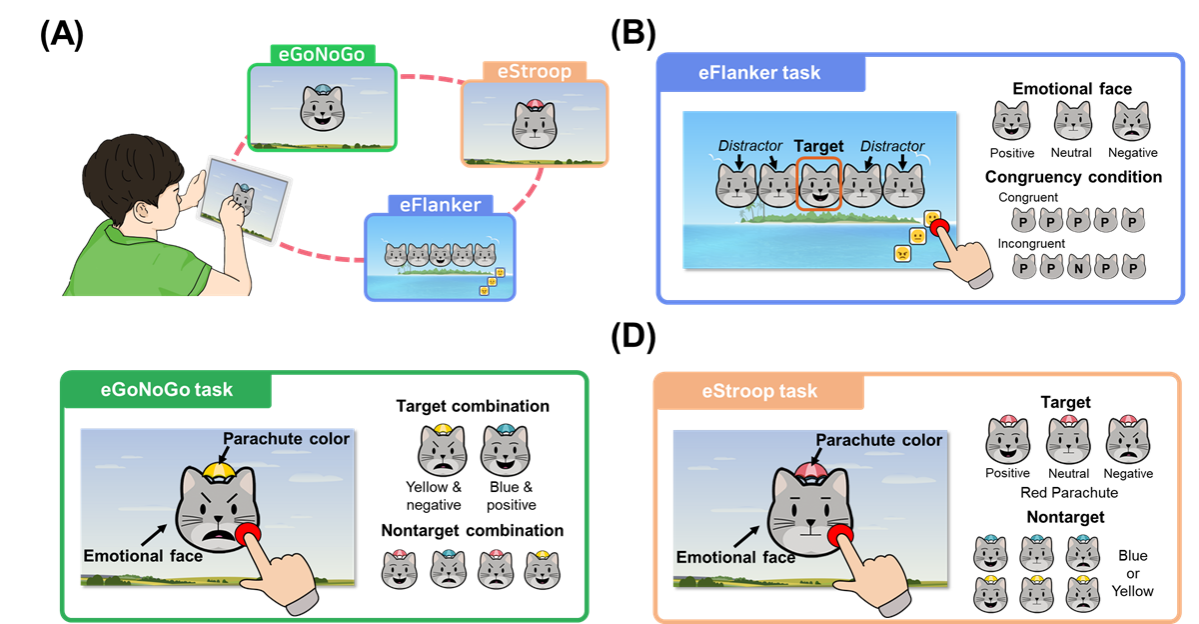
(A) The 3 emotional cognitive tasks used in this study. A diagram with an example screenshot of the emotional cognitive tasks: (B) emotional Flanker task (eFlanker), (C) emotional Go/No-Go task (eGoNoGo), and (D) emotional Stroop task (eStroop).

We designed 3 emotional cognitive tasks (eFlanker, eGoNoGo, and eStroop) to measure attention, working memory, and selective inhibition along with emotional processing (Figure 1). All tasks were developed based on the PsychoPy software (version 23.1.2; Open Science Tools Ltd) and implemented on a computer with responses recorded using a touchscreen monitor.

#### eFlanker

The eFlanker was designed to test for attention and its interactions with emotional stimuli [43,44]. Children were instructed to choose the face (located at the bottom right side of the screen) expressing the same emotion as the target, which was presented in the middle, with 2 distractors on both sides of the wings. The target and distractors were presented after a brief fixation cross (1000 ms), and feedback was provided on the screen once the child responded. The target and distractor consisted of 3 emotional (positive, neutral, and negative) cat faces, and there were a total of 9 possible combinations for their presentation as the target and distractor. Children performed 18 trials of practice blocks to familiarize themselves with the task, and then conducted 2 main blocks with 90 trials each (total 180 trials, 20 trials for each condition), with trial types presented in a random order. A break time was given between the main blocks.

#### eGoNoGo

The eGoNoGo was designed to measure emotional bias in executive function and memory by testing one’s ability to choose the target stimuli while withholding responses to the nontarget objects [45,46]. Out of 6 possible combinations from 3 parachute colors (yellow, red, and blue) and 2 emotions (positive and negative), children were instructed to respond to 2 target combinations of a specific parachute color and a specific emotional cat face (eg, blue parachute with the positive cat face and the yellow parachute with the negative cat face). The color-emotion combination was counterbalanced and randomly assigned across participants. After the fixation cross was presented for 1000 milliseconds, a stimulus was presented in the middle of the screen. The nontarget stimuli were presented for 1500 milliseconds, and the target stimuli were presented until the participants responded. If participants responded to the nontarget (incorrect response) or responded later than 2000 milliseconds, visual feedback on their performance (“Incorrect” or “Respond quickly”) was displayed for 1000 milliseconds. Children performed 20 practice trials and then 2 main blocks with 60 trials each (total 120 trials). We adjusted the frequency of the target trials to be slightly higher, to 40% of the total trials, to offset the imbalance between the target (2 out of 6 possible combinations) and nontarget trials (4 out of 6) to keep the children engaged in the task. A break time was given between the main blocks.

#### eStroop

The eStroop was designed to test inhibitory control by regulating children’s response to the nontarget object. Children were asked to touch the face stimuli with a specific parachute color (either red, blue, or yellow) while ignoring the other colors, regardless of the emotion of the face (positive, neutral, and negative). The color-emotion combination was counterbalanced across participants. After a fixation cross was presented for 1000 milliseconds, the nontarget trial was presented for 2500 milliseconds, and the target trial was presented until the participants responded. If participants responded to the nontarget (incorrect response) or responded to the target later than 2000 milliseconds, visual feedback (“Incorrect” or “Respond quickly”) was displayed for 1000 milliseconds. Children performed 12 practice trials and then 3 main blocks with 36 trials each (total 108 trials). A break time was given between the main blocks.

### Procedures

The experiments were conducted in a quiet room either in the laboratory or in the local children’s museum. Both experimental environments were child-friendly and were arranged to be similar to each other. Anxiety and depression levels were measured using the State-Trait Anxiety Inventory for Children (STAI-CH) [47] and Center for Epidemiological Studies Depression Scale for Children (CES-DC) [48] scales, which were designed specifically for children. In addition, their caregivers completed the ADHD questionnaire using the K-ARS [49] based on *DSM-5* (*Diagnostic and Statistical Manual of Mental Disorders* [Fifth Edition] [50]).

Children and their caregivers were provided with informed consent and a description of the overall task. After a brief instruction on the tasks, followed by practice sessions, the children performed the 3 emotional cognitive tasks in a mixed order. During this time, the caregivers completed the ADHD survey (K-ARS) about their children. After the completion of the main tasks, the children were surveyed on questions about their mental health (CES-DC and STAI-CH) either through a written form on an electronic device or through conversation with the experimenter, based on their preference. We did not observe significant differences in CES-DC or STAI-CH scores between the self-completed and experimenter-administered responses (CES-DC: t_73_=–0.725; *P*=.47; STAI-CH: t_73_=0.09; *P*=.93; 2-sample *t* test).

### Missing Data

Some children missed 4 or more consecutive trials due to distraction or an explicit request to discontinue participation during the task. In total, 14 children met these criteria. Specifically, the number of missing cases was 2 for the eFlanker, 4 for the eGoNoGo, and 10 for the eStroop. These data were excluded from the entire analysis.

### Behavioral Indices from the Emotional Cognitive Tasks

Accuracy and RT were calculated from the emotional cognitive tasks to compare behavioral performance across conditions and children. We averaged RTs from the correct trials, removing those shorter than 200 milliseconds to eliminate accidentally pressed responses. Outliers for RT or accuracy in each emotional condition were excluded at the subject level. Outliers were defined as values exceeding 3 times the median absolute deviation from the median, where the median absolute deviation was computed as the median of the absolute deviations from the median.

### Extracting E-Scores as an Abstract Measure of Task Performance

Abstract scores were required to summarize a range of behavioral indices in the children’s performance on the emotional cognitive tasks. The behavioral indices from 64 children were used for these analyses, after removing 14 children who lost interest during 1 of the 3 tasks (missing data), resulting in RT or accuracy values that were not appropriate for measuring an association with their mental health or ADHD. First, we calculated 82 behavioral indices consisting of RT and accuracy from various conditions of the 3 tasks (eFlanker, eGoNoGo, and eStroop), including averaged RT/accuracy of whole trials or emotion-related RT/accuracy difference between emotional conditions or between congruent and incongruent conditions (Table S1 in Multimedia Appendix 1). Second, to extract the most informative components while minimizing collinearity, 2D latent features were derived by extracting the first and second principal components (PCs) of the *z-*scored behavioral indices [33,51,52]. To avoid having the principal component analysis (PCA) dominated by the overrepresentation of outliers, *z*-scored indices with an absolute value over 2.5 were reduced to 2.5. Only the first two PCs explained more than 10% of the variance (Figure S1 in Multimedia Appendix 1). Then, the k-means clustering method was applied to the 2 PCs for dividing the latent features into several groups based on their squared Euclidean distance [53,54]. The optimal number of groups to maximally separate the latent features was determined by identifying the point at which the rate of decrease in inertia (within-cluster sum of squared errors) slowed down [55] (Figure S1 in Multimedia Appendix 1). Behavioral indices within the same group represented a similar pattern across the participants (eg, RT in the eFlanker was highly correlated with RT in the eStroop). Lastly, we calculated E-scores ranging from 1 to 3 by calculating the first PC of *z*-scored behavioral indices from each of the 3 groups.

### Statistical Tests

Behavioral performance across emotional conditions was compared using paired *t* tests with a 2-sided significance level of α=.05. We also performed analysis of covariance (ANCOVA) on task performance measures (RT and accuracy) and E-scores, with gender as a fixed factor and age as a continuous covariate. Pearson partial correlation analyses were conducted to assess associations between E-scores and mental health or ADHD scales while controlling for age.

## Results

### Depression, Anxiety, and ADHD Scales Across Development

We investigated whether children’s anxiety, depression, and ADHD scores were correlated with one another and how they differed across age. First, there was a strong association between depression (CES-DC) and anxiety (STAI-CH; Pearson correlation *r*=0.493; *P*<.001), while the risks for ADHD (K-ARS) were not correlated with either anxiety or depression (K-ARS and CES-DC: *r*=0.068; *P*=.58; K-ARS and STAI-CH: *r*=0.062; *P*=.61). When we conducted an ANCOVA with gender as a fixed factor and age as a covariate, we found higher depression symptoms in younger (*F*_1,72_=7.044; *P*=.01) and male children (*F*_1,72_=5.822, male 95% CI 12.8-19, female 95% CI 9.79-13.4; *P*=.02). To avoid the possibility that the CES-DC and STAI-CH self-report scales was too difficult for 1 child aged <5 years, we conducted the same analysis without that child and found that the effect of age on the depression symptoms still showed a strong trend but was not significant (*F*_1,71_=3.495; *P*=.07), while the gender effect was still significant (*F*_1,71_=4.68; *P*=.03). Self-reported anxiety and parent-reported ADHD levels were not significantly different across age and gender (anxiety: age effect *F*_1,71_=0.628; *P*=.43 and gender effect *F*_1,71_=0.009; *P*=.93; ADHD: age effect *F*_1,70_=0.719; *P*=.4 and gender effect *F*_1,70_=0.001; *P*=.99).

### Emotion-Related Effects in Emotional Cognitive Task Performance

**Figure 2:**
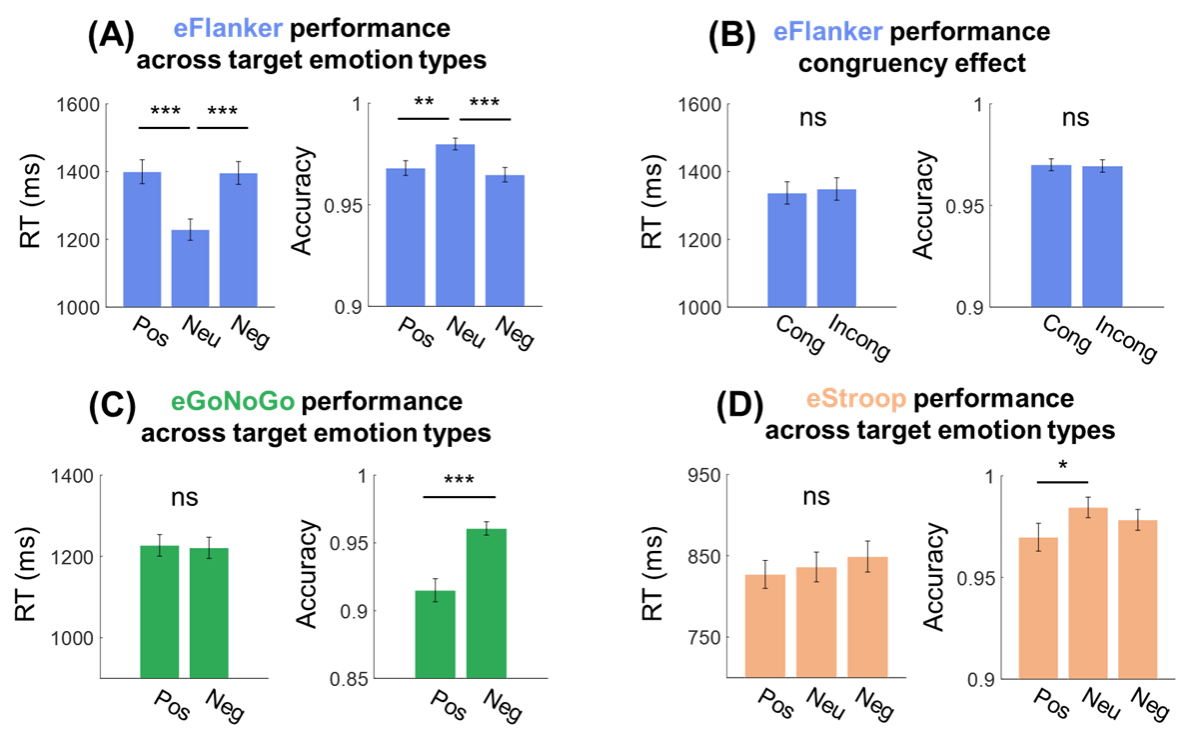
Behavioral results (reaction time [RT] and accuracy) from all participants in the 3 emotional cognitive tasks. (A) RT (left) and accuracy (right) across 3 emotional conditions (positive [Pos]; neutral [Neu]; and negative [Neg]) in the emotional Flanker task (eFlanker). (B) RT and accuracy in congruent (Cong) and incongruent (Incong) conditions in the eFlanker. (C) RT and accuracy across positive and negative conditions in the emotional Go/No-Go task (eGoNoGo). (D) RT and accuracy across positive, neutral, and negative conditions in the emotional Stroop task (eStroop). Error bars indicate SE. ns: not significant. *P<.05, **P<.01, ***P<.001.

We found a strong emotion-related effect on children’s attention in the eFlanker (Figure 2A). First, RT was significantly higher in both positive (95% CI 1330-1469 ms) and negative (95% CI 1329-1463 ms) conditions compared to the neutral (95% CI 1167-1290 ms) condition (positive vs neutral t_68_=11.404; *P*<.001; negative vs neutral: t_67_=9.821; *P*<.001). Similarly, accuracy was lower for the 2 emotional conditions (positive: 95% CI 0.961-0.975; negative: 95% CI 0.958-0.972) than the neutral condition (95% CI 0.974-0.986; positive vs neutral: t_63_=3.414; *P*=.001; negative vs neutral: t_65_=5.859; *P*<.001), indicating that children struggled to suppress the distraction induced by emotional stimuli. However, there were no significant differences between congruent and incongruent conditions in both RT (congruent: 95% CI 1272-1402 ms; incongruent: 95% CI 1284-1414 ms) and accuracy (congruent: 95% CI 0.964-0.976; incongruent: 95% CI 0.963-0.976) on the eFlanker (Figure 2B; RT: t_68_=0.265; *P*=.79; accuracy: t_64_=0.331; *P*=.74). This lack of a significant congruency effect may be attributed to the dominant effects of emotional face stimuli, as reported in a previous study [56]. In the eGoNoGo, children made significantly more errors in the positive condition (positive: 95% CI 0.898-0.932; negative: 95% CI 0.951-0.97; t_59_=4.776; *P*<.001; Figure 2C), which was possibly a result of failure to inhibit a response to the rewarding stimuli. RT was not different between positive and negative conditions (positive: 95% CI 1175-1300 ms; negative: 95% CI 1170-1273 ms). Similarly, in the eStroop, accuracy in the positive condition (95% CI 0.956-0.983) was significantly lower compared to the neutral condition (95% CI 0.975-0.994; t_67_=2.6; *P*=.01; Figure 2D), while RT was not different across emotion (positive: 95% CI 793-861 ms; negative: 95% CI 812-886 ms) and neutral conditions (95% CI 800-872 ms).

### Emotional Cognitive Task Performance Across Development

**Figure 3:**
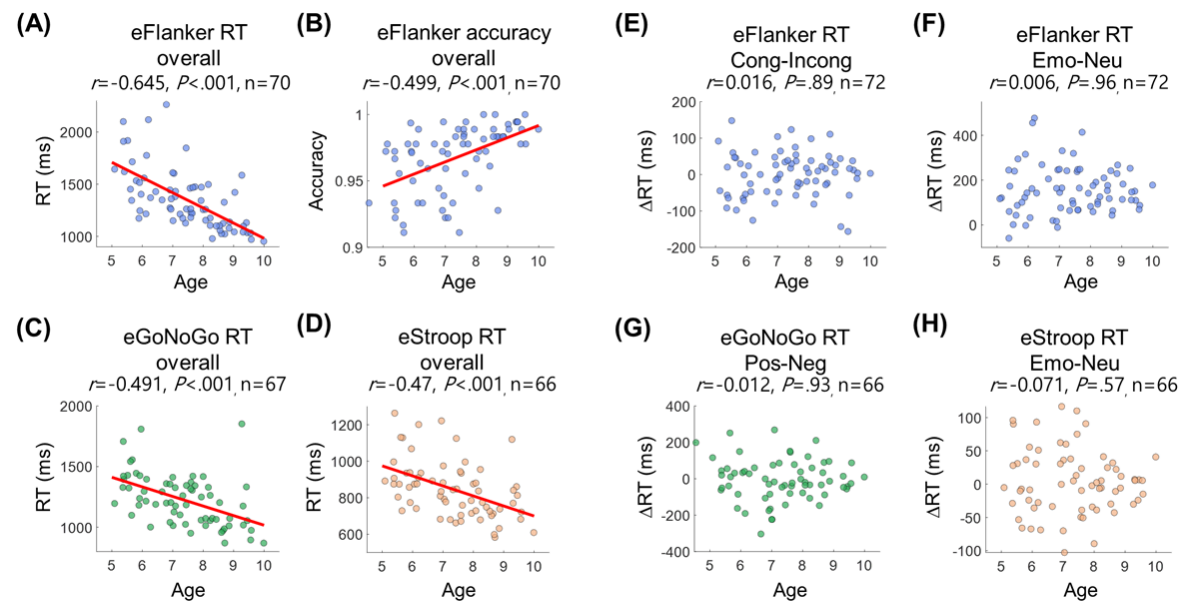
Developmental changes in overall performance, congruency effect, and emotional sensitivity were examined using a cross-sectional dataset. (A-D) Scatter plots showing significant relationships between age and performance in the 3 emotional cognitive tasks. (E-H) The congruency effect in the emotional Flanker task (eFlanker) and emotional sensitivity in reaction time (RT) across all tasks were not correlated with age. Cong: congruent; eGoNoGo: emotional Go/No-Go task; Emo: emotional; eStroop: emotional Stroop task; Incong: incongruent; Neg: negative; Neu: neutral; Pos: positive.

ANCOVA tests were conducted on general task performance measured by RT and accuracy with gender as a fixed factor and age as a covariate. General task performance in all tasks was strongly correlated with age, confirming an improvement in attention-related functions between age 4 and 10 years (eFlanker RT: *F*_1,66_=50.817; *P*<.001; accuracy: *F*_1,66_=22.349; *P*<.001; eGoNoGo RT: *F*_1,63_=19.513; *P*<.001; accuracy: *F*_1,62_=8.281; *P*=.005; eStroop RT: *F*_1,62_=16.585; *P*<.001; accuracy: *F*_1,61_=5.879; *P*=.02; see Pearson correlation coefficients in Figures 3A-3D). We found a faster RT in male children in the eStroop RT (male: 95% CI 765-843 ms, female: 95% CI 838-949 ms; *F*_1,62_=4.933; *P*=.03), but no gender differences were found in the other behavioral measures.

The congruency effect (ie, RT and accuracy differences between congruent and incongruent conditions) or performance difference between emotional and neutral targets in the eFlanker did not change in this age range (Figures 3E and 3F). Additionally, the RT or accuracy difference between positive and negative conditions in the eGoNoGo did not show any changes across age (Figure 3G). Similarly, the difference in eStroop performance (RT and accuracy) between the emotional and neutral conditions was not correlated with age (Figure 3H). None of these behavioral indices showed between-gender differences.

### Relationship Between Emotional Cognitive Task Performance and Mental Health Measures

**Figure 4:**
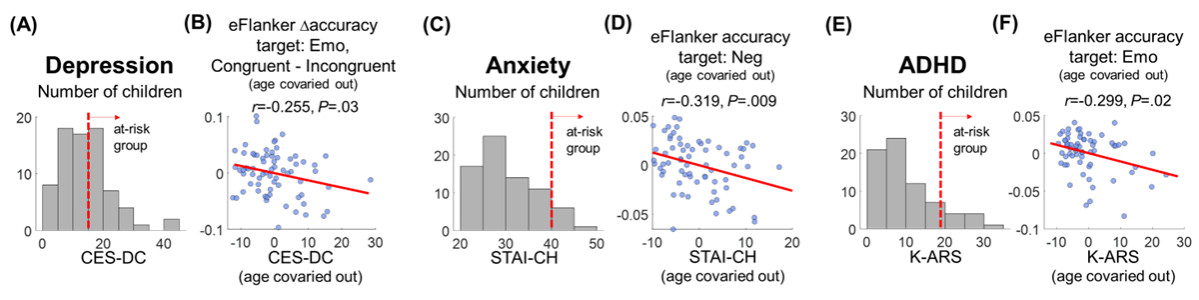
Relationship between task performance and mental health/attention-deficit/hyperactivity disorder (ADHD) scales. (A) Distribution of children’s self-reported depression scales (Center for Epidemiological Studies Depression Scale for Children [CES-DC]). (B) Correlation between the CES-DC scale and the congruency effect on the emotional Flanker task (eFlanker) accuracy after covarying out age. A lower congruency effect was observed in children with a higher CES-DC level. (C) Distribution of children’s self-reported anxiety scales (State-Trait Anxiety Inventory for Children [STAI-CH]). (D) Lower eFlanker accuracy in the negative (Neg) condition was associated with a higher STAI-CH level. (E) Distribution of children’s ADHD scales, based on parent reports (Korean ADHD Rating Scale [K-ARS]). (F) A lower eFlanker accuracy in the emotional (Emo) condition was observed in children with a higher K-ARS level.

Among the children recruited for this study, 28 (36%) children (CES-DC >15 [57]) were in the high-risk group of depression based on the CES-DC scale (Figure 4A). Depressed mental health was associated with emotional processing during the eFlanker, as RT difference between emotion and neutral target conditions was higher for children with a higher depression scale (Pearson correlation between CES-DC and ∆RT emotional-neutral: *r*=0.281; *P*=.02 after controlling for age; Table 1). In addition, the congruency effect on accuracy (ie, a higher accuracy in the congruent condition compared to the incongruent condition) in the eFlanker was reduced in children with higher CES-DC (Figure 4B; *r*=–0.255; *P*=.03; n=70).

**Table 1:** Behavioral indices derived from the emotional cognitive tasks, grouped according to principal component analysis (PCA), with coefficients for E-score calculation. Only indices that showed a significant Pearson correlation with mental health measures (Center for Epidemiological Studies Depression Scale for Children [CES-DC] and State-Trait Anxiety Inventory for Children [STAI-CH]) or attention-deficit/hyperactivity disorder symptoms (Korean ADHD Rating Scale [K-ARS]) after covarying out age or overall task reaction time/accuracy on each task are presented. The complete lists are provided in Table S1 in Multimedia Appendix 1.

Group	Task	Feature	PCA coefficient	Age, *r*	CES-DC, *r*^a^	STAI-CH, *r*^a^	K-ARS, *r*^a^
1	eFlanker^b^	Overall reaction time	0.201	–0.645^c^	0.106	–0.019	–0.131
1	eFlanker	Reaction time (negative target, neutral distractor)	0.188	–0.714^c^	0.237^c^	0.113	–0.101
1	eFlanker	Reaction time (negative target, negative distractor)	0.192	–0.667^c^	0.262^c^	–0.094	–0.062
1	eFlanker	Reaction time (emotional target, congruent distractor)	0.210	–0.632^c^	0.293^c^	0.034	–0.028
1	eGoNoGo^d^	Overall reaction time	0.146	–0.491^c^	0.176	0.132	–0.119
1	eStroop^e^	Overall reaction time	0.137	–0.47^c^	0.039	0.174	–0.177
2	eFlanker	Reaction time difference (emotional vs neutral targets; all distractors)	0.267	0.006	0.282^c^	0.158	–0.053
2	eFlanker	Accuracy (negative target, negative distractor)	–0.211	0.232^c^	–0.397^c^	–0.354^c^	–0.108
2	eFlanker	Accuracy difference (positive vs neutral targets; same-valence distractors)	–0.180	0.105	–0.263^c^	–0.186	–0.090
2	eFlanker	Accuracy difference (negative vs neutral targets; same-valence distractors)	–0.220	0.015	–0.235^c^	–0.232	–0.059
2	eFlanker	Accuracy difference (emotional-congruent vs neutral-neutral trials)	–0.250	0.131	–0.303^c^	–0.223	–0.053
3	eFlanker	Overall accuracy	0.285	0.499^c^	–0.013	–0.167	–0.255^c^
3	eFlanker	Accuracy (emotional target, incongruent distractor)	0.273	0.390^c^	0.143	–0.014	–0.329^c^
3	eFlanker	Accuracy (positive target, incongruent distractor)	0.274	0.344^c^	–0.060	–0.003	–0.383^c^
3	eFlanker	Accuracy (negative target, incongruent distractor)	0.224	0.381^c^	–0.096	–0.218	–0.29^c^
3	eFlanker	Accuracy difference (negative targets; incongruent vs congruent distractors)	0.062	0.009	0.129	0.220	–0.249^c^
3	eFlanker	Accuracy difference (emotional targets; incongruent vs congruent distractors)	0.146	0.204	0.257^c^	0.218	–0.142
3	eFlanker	Accuracy (all targets, incongruent distractor)	0.297	0.5^c^	0.034	–0.108	–0.333^c^
3	eFlanker	Accuracy (emotional targets)	0.287	0.432^c^	–0.071	–0.125	–0.322^c^
3	eGoNoGo	Overall accuracy	0.188	0.333^c^	–0.078	0.036	–0.299^c^
3	eGoNoGo	Accuracy (positive target)	0.139	0.423^c^	–0.113	–0.018	–0.292^c^
3	eStroop	Overall accuracy	0.165	0.292^c^	0.072	0.091	–0.145

^a^Age was covaried when calculating the Pearson correlation coefficients.

^b^eFlanker: emotional Flanker task.

^c^Indicates a statistical significance with *P* value of <.05.

^d^eGoNoGo: emotional Go/No-Go task.

^e^eStroop: emotional Stroop task.

Compared to depression, the number of children in the moderate to high anxiety group was lower (n=7, 9%; Figure 4C; STAI-CH >39 [58]). Accuracy on the eFlanker in the negative condition was the only behavioral measure correlated with the STAI-CH scale (Figure 4D; *r*=0.319; *P*=.009 after controlling for age; n=67). This may be due to a lower number of children with high anxiety, resulting in less clear across-individual contrasts for each behavioral index.

Lastly, a total of 10 (12.8%) children (K-ARS >18 [59]) were in the high-risk group for ADHD (Figure 4E). These children showed less accurate performance during the eFlanker and eGoNoGo, especially in the emotional condition, compared to children with lower K-ARS scores (Figure 4F; *r*=–0.299; *P*=.02 after controlling for age; n=64).

### Extracting “E(motional)-Scores” Using Behavioral Indices From the Emotional Cognitive Tasks

**Figure 5:**
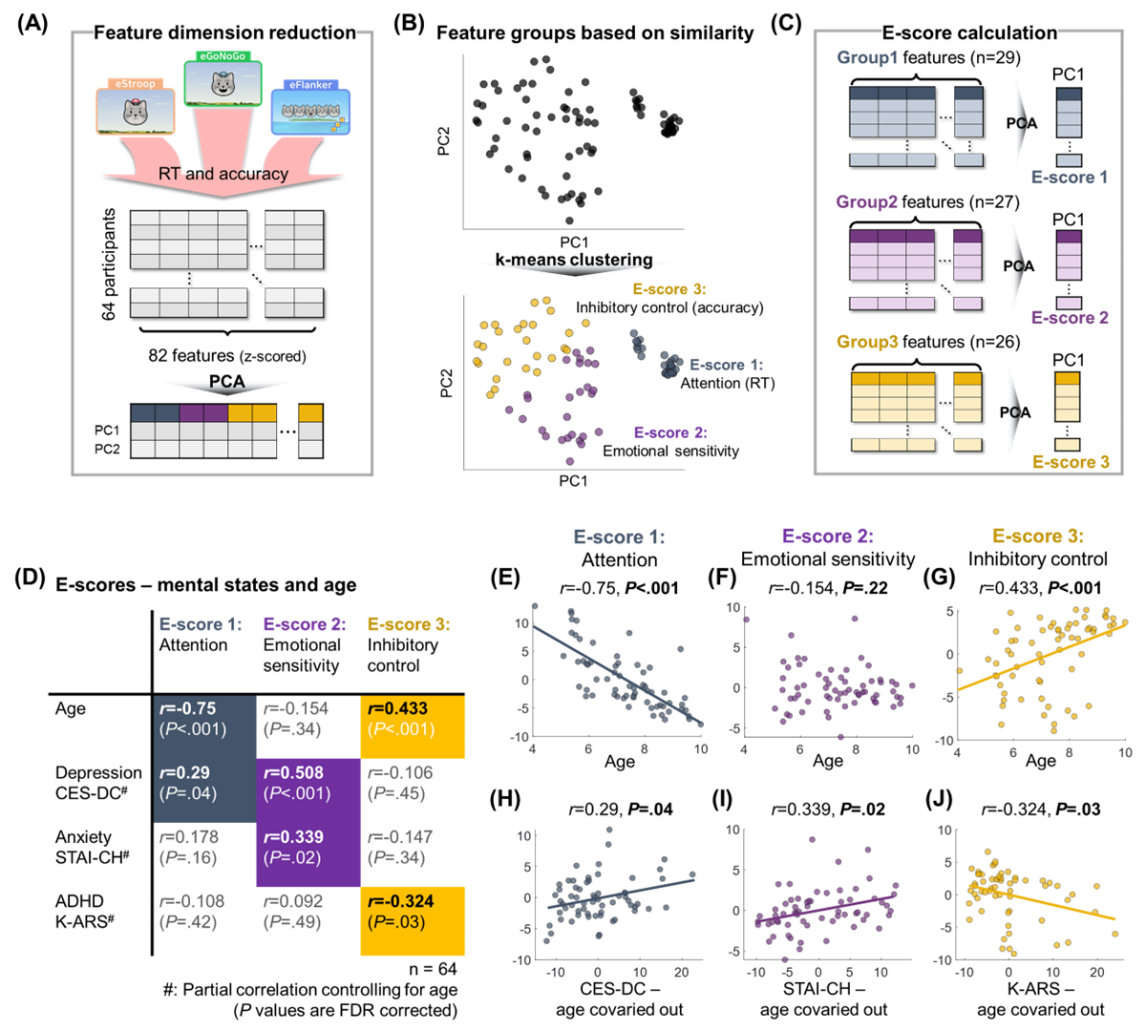
Grouping of features (general reaction time [RT] and accuracy, the effects of emotion, and congruency effects) to calculate E-scores. (A) Dimensionality reduction of features based on their similarity across participants by principal component analysis (PCA). (B) The result of feature grouping after k-means clustering visualized on a 2D plane for the first 2 principal components (PCs). (C) Calculating E-scores using the first PC from each feature group. (D) Relationship between children’s individual characteristics (ie, age, depression, anxiety, and attention-deficit/hyperactivity disorder [ADHD] scales) and E-scores. Only statistically significant (*P*<.05) linear relationships are visualized with a best-fit line. (E-G) Correlation between E-scores and age. (H-J) Correlation between each E-score and mental health/ADHD scales after controlling for age. CES-DC: Center for Epidemiological Studies Depression Scale for Children; FDR: false discovery rate; K-ARS: Korean ADHD Rating Scale; STAI-CH: State-Trait Anxiety Inventory for Children.

Given the nature of the tasks and the age of the participants, each behavioral index showed high variance across trials. In addition, we observed high redundancy as many behavioral measures were highly correlated with one another. Therefore, we opted to extract the common pattern spread over many behavioral indices, while maintaining the amount of information. We applied dimensionality reduction to the behavioral indices from the 3 emotional cognitive tasks to extract condensed information of individual performance by reducing repetitiveness (Figure 5A). Behavioral features (*D*=82) were divided into 3 groups by applying the k-means clustering method (Figure 5B), and E-scores were calculated by extracting the first PC of behavioral indices from each group (Figure 5C). We confirmed the robustness of the clustering using leave-one-out cross-validation, which assigned each excluded sample to the nearest group center and yielded labels identical to the original grouping.

The first feature group contained RT features in various conditions across the eFlanker, eGoNoGo, and eStroop, thus representing the attention function (see Table S1 in Multimedia Appendix 1 for the whole list of behavioral features and coefficients for the first PC calculation). Positive PC coefficients of RT features for E-score 1 indicated that a higher E-score 1 represented decreased attention. Next, the second feature group represented complex interactions between emotion and attention. Coefficients of the first PC indicated that a higher E-score 2 was associated with a longer RT and lower accuracy, particularly in the emotional conditions, as well as higher congruency effects on RT in the eFlanker. Additionally, longer RT in the negative condition of the eStroop compared to the neutral condition also contributed to E-score 2. The third feature group represented accuracy on the tasks, as coefficients of accuracy features across various conditions in all 3 tasks positively contributed to E-score 3. Additionally, higher accuracy in the incongruent condition (ie, lower congruency effect) of the eFlanker also contributed to E-score 3.

### Relationships Between E-Scores and Depression, Anxiety, or ADHD Symptoms

We evaluated the relevance of E-scores as individual emotional-cognitive characteristics by examining their correlations with mental health and ADHD survey scales (Figure 5D). First, ANCOVA tests were applied on E-scores with gender as a fixed factor and age as a continuous covariate. E-scores 1 and 3, which represented attention and executive function based on RT and accuracy, were significantly associated with age (E-score 1: *F*_1,60_=78.404; *P*<.001; E-score 3: *F*_1,60_=14.673; *P*<.001; all *P* values here and below are corrected for false discovery rate; see Pearson correlation coefficients in Figures 5E and 5G). In contrast, E-score 2, representing emotion-attention interactions, was not correlated with age (Figure 5F; *F*_1,60_=1.493; *P*=.23). None of the E-scores showed significant between-gender differences. Self-reported depression level measured by the CES-DC was associated with E-score 1 (Figure 5H; *r*=0.29; *P*=.04), which replicated previous studies showing that children with depressive symptoms showed lower attention function represented by a longer RT [60]. In addition, a significant positive correlation between the CES-DC scale and E-score 2 (*r*=0.508; *P*<.001) indicated that children with higher depression were more sensitive to the emotional stimuli, resulting in longer RT and lower accuracy. E-score 2 was also positively correlated with the anxiety scale (ie, STAI-CH; Figure 5I; *r*=0.339; *P*=.02), showing similar cognitive characteristics induced by anxiety and depression. No differences in these association patterns were observed between the 2 collection methods (self-completed vs experimenter-guided; see Table S2 in Multimedia Appendix 1 for details). Lastly, E-score 3, which represented accuracy, was negatively correlated with the ADHD scale (ie, K-ARS; Figure 5J; *r*=–0.324; *P*=.03), suggesting that children with a higher risk for ADHD were more impulsive and inaccurate in their responses to emotional stimuli.

To further disentangle the effects of depression, anxiety, and ADHD on task performance (ie, E-scores), we conducted partial correlation analyses by controlling for the other scales and age (Table S3 in Multimedia Appendix 1). Every correlation remained significant except for E-score 2 and STAI-CH, which suggested that the E-score 2 does not reflect an anxiety-specific effect but rather overlapping characteristics shared between the anxiety and depression measures.

The dimensionality reduction technique revealed common and distinct behavioral patterns across the 3 emotional cognitive tasks. Extracting the common patterns contributed to reducing noise in the behavioral data and thus compensated for the short performance time of the tasks (10-15 minutes for each task), while the distinctiveness contributed to collecting various affective-cognitive profiles of children. Therefore, a range of behavioral indices was reduced to condense features, which were translated into a more robust, composite measure for individual affective-cognitive characteristics that is not susceptible to biases in a single measure. The results highlight the dissociability across depression, anxiety, and ADHD risk, given that each scale was correlated with different E-scores.

## Discussion

### Principal Findings

In this study, we aimed to investigate the distinct impacts of depression, anxiety, and ADHD on emotion recognition and attention in children aged between 4 and 10 years, using child-friendly gamified eFlanker, eGoNoGo, and eStroop. Our results highlight that various task performance measures related to emotion and cognition can be summarized into three distinctive components (attention, selective inhibition, and emotional sensitivity). Based on this, we introduced E-scores to incorporate meaningful information about the participant while minimizing noisy data distributed across the various features. The E-scores provided enhanced interpretability of behavioral task performance and were associated with mental health (anxiety and depression) or ADHD symptoms. The emotional cognitive assessments of our study are advantageous in that they allow monitoring of children’s neurocognitive development, which may help address current limitations of measurement through self-report surveys.

### Comparison to Prior Literature

The main novelty of this study lies in the development and validation of digital tasks that measure interactions between individual cognitive and affective characteristics and their associations with age-, mental health–, and ADHD-related measures within a unified framework. This approach offers advantages over previous studies that focused on single symptom domains [22-24], as it facilitates interpretation of children’s behavioral outcomes while reducing potential confounds arising from comorbidity. Administering 3 digital cognitive tasks across multiple emotional conditions generated a wide range of behavioral indices; to address the resulting analytical complexity, we applied dimensionality reduction techniques to extract informative features across these indices. Together, these differences in study aims and methodological approach distinguish this work from prior studies.

As reported in many other studies [61], we observed a high correlation between depression and anxiety symptoms in our sample. Both anxiety and depression were associated with a larger emotional interference in attention functions (ie, RT increase and accuracy decrease in the emotional compared to the neutral conditions), typically higher for negative stimuli (Figure 4D). This is consistent with our previous study in which emotional bias served as a marker of both depression and anxiety [33]. Anxious and depressed individuals often show decreased inhibitory control [62] and higher emotional sensitivity [63], and thus they have a higher chance of being distracted by emotional stimuli [33,64]. This pattern has been linked to a hyperactive amygdala, which is also associated with weaker top-down control and reduced activation in the dorsolateral PFC and ventromedial PFC [65,66].

ADHD risk measured by the K-ARS scale survey was independent of the depression or anxiety scales. Similarly, unlike depression or anxiety, ADHD was strongly associated with accuracy across various conditions in the 3 tasks. Children with ADHD have difficulty inhibiting irrelevant stimuli, reflecting deficits in selective attention and executive control [67]. The results in the eFlanker suggested that children with higher K-ARS scores showed reduced selective inhibition toward emotional stimuli (compared to neutral ones), which is consistent with their higher impulsivity [68,69]. Similarly, the lower accuracy observed in the eGoNoGo and eStroop likely resulted from disrupted working memory and executive control due to higher impulsivity. In particular, the general tendency for young children to respond to an incorrect positive stimulus in the eGoNoGo and eStroop are consistent with that interpretation. Interestingly, children’s ADHD score was only associated with accuracy and not RT; this may be related to the fact that impulsive individuals tend to prioritize speed over accuracy, according to the well-known speed-accuracy trade-off, especially in the Flanker task [70].

Previously, we found that children’s performance in the emotional cognitive tasks can be characterized as longer RT and lower accuracy compared to adults [33]. In this study, a cross-sectional investigation with a wider range of age showed that older children performed better than younger children, indicating that their general attention functions develop across childhood (4-10 years of age) with the maturation of the PFC [71]. Another characteristic of the emotional cognitive tasks is a general tendency for longer RT and lower accuracy in the emotional conditions. In particular, children exhibited more errors for positive stimuli than for neutral or negative stimuli, likely reflecting greater attentional bias toward positive cues, which can be associated with stronger approach motivation and heightened reward sensitivity [72]. However, these emotion-attention interactions remained unchanged across ages in our study. Previous studies reported that the emotional sensitivity of children is relatively higher until the age of around 10 years, when the amygdala reactivity decreases along with PFC maturation [73,74]. Thus, it is possible that all children in our study were significantly affected by emotional stimuli.

One difference between the results of our eFlanker with young children and our previous study [33] with adults is the reduction in the congruency effect. This discrepancy can be explained by developmental attention mechanisms that describe children’s attentional bias toward more salient or emotional stimuli [7,8]. Congruency effects, characterized by longer RT and lower accuracy in the incongruent conditions, are induced by increased cognitive resources needed to resolve conflict in the incongruent condition and are typically accompanied by increased activity in the anterior cingulate cortex and other frontal regions [75]. However, as emotional targets are likely to attract more attention in children, flankers may be equally distracting in both congruent and incongruent conditions. Consistent with this interpretation, a reduced or absent congruency effect has also been reported in a previous study using a complex Flanker task with emotional faces as either target or distractor stimuli [56].

### Strengths

The prevalence of childhood depression and ADHD has drastically increased over the past few years [76,77], and thus, a daily monitoring tool is essential for risk detection within the optimal time window for early intervention. We demonstrated that a simple emotional cognitive assessment tool can provide a stronger scientific basis for identifying affective-cognitive characteristics related to mental health and ADHD. Our approach highlights the efficacy of simple and easy, child-friendly, touchscreen-based tasks that are enjoyable even for young children, which is a critical factor for inducing accurate measurement of their attention, emotional sensitivity, and inhibitory function [78,79]. Furthermore, the average duration of gameplay was around 30 minutes, which is manageable and sustainable for repeated testing in children. These advantages of our tool enable an easy but quantifiable measurement of cognitive symptoms related to children’s mental health and ADHD symptoms (eg, during school activities) for parents and clinicians.

### Limitations and Future Directions

One limitation of this study is that it is based on a subclinical sample of children and that the mental health and ADHD measures were based on self-report and parent surveys. To enhance its applicability and generalizability to patient populations, future studies should validate the results using a formal clinical diagnosis. Nevertheless, in our previous study [33], we demonstrated that the relationships between task performance and mental health observed in healthy adults were also present in individuals diagnosed with depression. Second, parent-reported assessments of ADHD can also be subject to bias influenced by factors such as cultural context, parental mental health, and variability in the interpretation of symptoms. To mitigate this, we provided standardized instructions and guided administration to minimize variability in parental interpretation of symptom items. Third, while our age range was within the recommended period for the ADHD assessment, it may be relatively young for reliably assessing depression and anxiety, particularly for children aged <5 years. Therefore, our prediction based on behavioral performance may indicate a risk for later developmental stages. Fourth, cultural differences could influence emotion-cognition interactions in young children. Although this factor was minimized in this study, as all participants were Korean, cultural considerations may need to be considered when applying the tool internationally. Fifth, some children lost interest in the task during testing, which limited our ability to accurately assess their affective-cognitive characteristics. Including these data would likely weaken the observed associations between behavioral indices and mental health or ADHD survey scales; therefore, these cases were treated as missing data, resulting in a reduced sample size. For future applications in daily-life, such as deployment as a mobile or web-based application, online algorithms to detect loss of engagement will be necessary. In addition, appropriate real-time outlier detection and data-quality control procedures will be required to reliably capture users’ cognitive and affective characteristics. Lastly, a longitudinal study would be useful to investigate the effectiveness of the tool in assessing and predicting mental health and ADHD over an extended period, into adolescence.

### Conclusion

Our gamified digital monitoring tool offers a practical and objective way to connect individual behavioral profiles with their affective and cognitive health, particularly for young children who may benefit greatly from early intervention but are difficult to accurately assess using traditional clinical interviews. While requiring further standardization and a well-established protocol to ensure data privacy, the ability to perform a rapid assessment also makes this tool well-suited for practical use in a variety of settings.

## Appendix

Multimedia Appendix 1 Additional figure and tables.

## Abbreviations

ADHD: attention-deficit/hyperactivity disorder
ANCOVA: analysis of covariance
CES-DC: Center for Epidemiological Studies Depression Scale for Children
DSM-5: Diagnostic and Statistical Manual of Mental Disorders (Fifth Edition)
eFlanker: emotional Flanker task
eGoNoGo: emotional Go/No-Go task
eStroop: emotional Stroop task
K-ARS: Korean ADHD Rating Scale
PC: principal component
PCA: principal component analysis
PFC: prefrontal cortex
RT: reaction time
STAI-CH: State-Trait Anxiety Inventory for Children

## Data Availability

The data that support the findings of this study are publicly available on GitHub [80].
