# Supplementary material for: A Digital Tool for Assessing the Distinct Effects of Depression, Anxiety, and Attention-Deficit/Hyperactivity Disorder (ADHD) on Children’s Emotional Cognitive Bias: Cross-Sectional Study
Source: J Med Internet Res. 2026 Feb 25;28:e86286. doi: 10.2196/86286 (PMC12980069; doi:10.2196/86286)
Supplement: Multimedia Appendix 1 [file jmir_v28i1e86286_app1.pdf]

Table S1. A list of behavioral indices from the emotional cognitive tasks and their grouping results along with coefficients for calculating the first principal component score, ANCOVA results (F-values), Pearson correlation coefficients. Only gender effects were measured by ANCOVAs with gender as a fixed factor and age as a continuous covariate while the other effects were measured by (partial) Pearson correlations.

Group	Task	Feature	Coefficients	Age	Gender	CES-DC	CES-DC (age out#)	STAI-CH	STAI-CH (age out#)	K-ARS	K-ARS (age out#)
1	eFlanker	RT overall	0.201	**-0.645**	2.858	0.208	0.106	-0.038	-0.019	-0.042	-0.131
1	eFlanker	RT T: pos / D: pos	0.216	**-0.537**	0.09	0.209	0.149	-0.07	-0.012	0.043	-0.023
1	eFlanker	RT T: pos / D: neu	0.19	**-0.651**	3.269	**0.358**	**0.24**	0.107	0.097	0.037	-0.022
1	eFlanker	RT T: pos / D: neg	0.192	**-0.606**	3.824	**0.262**	0.171	0.068	0.07	0.033	-0.033
1	eFlanker	RT T: neu / D: pos	0.192	**-0.697**	0.971	0.165	0.035	0.062	0.074	0.021	-0.078
1	eFlanker	RT T: neu / D: neu	0.209	**-0.67**	1.21	0.221	0.108	0.004	0.031	-0.026	-0.122
1	eFlanker	RT T: neu / D: neg	0.193	**-0.678**	3.89	0.169	0.042	0.027	0.028	0.007	-0.074
1	eFlanker	RT T: neg / D: pos	0.198	**-0.634**	0.996	0.227	0.133	-0.062	-0.051	-0.063	-0.156
1	eFlanker	RT T: neg / D: neu	0.188	**-0.714**	1.205	**0.36**	**0.237**	0.13	0.113	-0.008	-0.101
1	eFlanker	RT T: neg / D: neg	0.192	**-0.667**	1.871	**0.321**	**0.262**	-0.11	-0.094	0.017	-0.062
1	eFlanker	RT T: emo / D: cong	0.21	**-0.632**	0.599	**0.353**	**0.293**	-0.007	0.034	0.047	-0.028
1	eFlanker	RT T: emo / D: incong	0.199	**-0.649**	2.466	**0.294**	0.211	0.002	0.025	0.007	-0.072
1	eFlanker	RT T: pos / D: incong	0.2	**-0.587**	3.717	0.223	0.134	-0.042	-0.025	0	-0.065
1	eFlanker	RT T: neu / D: incong	0.197	**-0.691**	3.204	0.159	0.031	-0.044	-0.026	-0.011	-0.101
1	eFlanker	RT T: neg / D: incong	0.203	**-0.657**	1.35	**0.265**	0.181	-0.016	0.011	-0.052	-0.146
1	eFlanker	RT T: all / D: cong	0.206	**-0.654**	1.501	**0.307**	0.228	0.059	0.104	0	-0.086
1	eFlanker	RT T: all / D: incong	0.205	**-0.665**	2.954	0.221	0.121	-0.037	-0.016	-0.025	-0.113
1	eFlanker	RT T: pos / D: all	0.207	**-0.599**	2.043	**0.303**	0.225	0.051	0.085	0.036	-0.029
1	eFlanker	RT T: neu / D: all	0.204	**-0.698**	2.087	0.213	0.093	0.001	0.028	0.006	-0.087
1	eFlanker	RT T: neg / D: all	0.2	**-0.68**	1.607	**0.288**	0.217	-0.092	-0.069	-0.016	-0.11
1	eFlanker	RT T: emo / D: all	0.2	**-0.623**	2.615	**0.248**	0.161	-0.018	0.007	-0.032	-0.112
1	eGoNoGo	RT overall	0.146	**-0.491**	2.253	**0.247**	0.176	0.158	0.132	-0.059	-0.119
1	eGoNoGo	RT pos	0.14	**-0.529**	1.091	**0.243**	0.167	0.134	0.103	-0.022	-0.081
1	eGoNoGo	RT neg	0.141	**-0.601**	2.268	**0.254**	0.176	0.144	0.104	-0.078	-0.135
1	eStroop	RT overall	0.137	**-0.47**	**4.933**	0.128	0.039	0.206	0.174	-0.089	-0.177
1	eStroop	RT pos	0.145	**-0.461**	1.074	0.202	0.126	0.175	0.133	-0.069	-0.149
1	eStroop	RT neu	0.137	**-0.419**	3.201	0.109	0.03	**0.247**	0.214	-0.084	-0.156
1	eStroop	RT neg	0.138	**-0.46**	**7.494**	0.109	0.019	0.226	0.197	-0.15	-0.245
1	eStroop	RT emo	0.139	**-0.469**	**5.113**	0.133	0.045	0.211	0.18	-0.101	-0.191
2	eFlanker	ΔRT (T:neg/D:neg) - (T:pos/D:pos)	0.095	-0.037	0.255	-0.05	-0.064	-0.173	-0.174	0.063	0.061
2	eFlanker	ΔRT (T:pos/D:pos) - (T:neu/D:neu)	0.127	**0.285**	0.447	-0.013	0.084	0.093	0.109	-0.016	0.017
2	eFlanker	ΔRT (T:neg/D:neg) - (T:neu/D:neu)	0.272	-0.045	0.548	0.206	0.202	-0.068	-0.068	0.023	0.012
2	eFlanker	ΔRT (T:emo/D:cong) - (T:neu/D:neu)	0.279	-0.04	0.278	0.211	0.207	0.048	0.047	0.053	0.038
2	eFlanker	ΔRT (T:pos/D:incong) - (T:pos/D:cong)	-0.168	-0.022	2.081	-0.045	-0.068	-0.202	-0.207	0.149	0.148
2	eFlanker	ΔRT (T:neu/D:incong) - (T:neu/D:cong)	-0.119	0.14	0.27	-0.066	-0.049	-0.044	-0.043	0.201	0.213
2	eFlanker	ΔRT (T:neg/D:incong) - (T:neg/D:cong)	-0.234	0.144	0.599	-0.191	-0.171	-0.004	-0.006	-0.122	-0.108
2	eFlanker	ΔRT (T:emo/D:incong) - (T:emo/D:cong)	-0.23	-0.052	0.001	-0.138	-0.151	-0.067	-0.067	-0.084	-0.089
2	eFlanker	ΔRT (T:all/D:incong) - (T:all/D:cong)	-0.243	0.016	0.088	-0.14	-0.147	-0.166	-0.165	0.066	0.069
2	eFlanker	ΔRT (T:pos/D:all) - (T:neu/D:all)	0.161	0.101	0.163	0.125	0.148	0.158	0.161	-0.005	0.004
2	eFlanker	ΔRT (T:neg/D:all) - (T:neu/D:all)	0.29	-0.034	0.72	0.177	0.174	0.115	0.114	-0.058	-0.068
2	eFlanker	ΔRT (T:emo/D:all) - (T:neu/D:all)	0.267	0.006	0.069	**0.274**	**0.282**	0.158	0.158	-0.049	-0.053
2	eFlanker	ACC T:pos / D: pos	-0.166	0.175	3.787	**-0.256**	-0.231	-0.095	-0.097	-0.142	-0.129
2	eFlanker	ACC T:neg / D: neg	-0.211	**0.232**	0.954	**-0.435**	**-0.397**	**-0.364**	**-0.354**	-0.124	-0.108
2	eFlanker	ACC T:emo / D: cong	-0.209	**0.3**	0.878	**-0.252**	-0.201	-0.233	-0.239	-0.095	-0.07
2	eFlanker	ΔACC (T:neg/D:neg) - (T:pos/D:pos)	-0.008	-0.098	1.91	0.008	-0.031	-0.065	-0.08	0.017	0.006
2	eFlanker	ΔACC (T:pos/D:pos) - (T:neu/D:neu)	-0.18	0.105	**4.362**	**-0.274**	**-0.263**	-0.193	-0.186	-0.098	-0.09
2	eFlanker	ΔACC (T:neg/D:neg) - (T:neu/D:neu)	-0.22	0.015	0.161	-0.23	**-0.235**	-0.233	-0.232	-0.054	-0.059
2	eFlanker	ΔACC (T:emo/D:cong) - (T:neu/D:neu)	-0.25	0.131	**7.196**	**-0.314**	**-0.303**	-0.23	-0.223	-0.064	-0.053
2	eFlanker	ΔACC (T:neu/D:incong) - (T:neu/D:cong)	0.016	-0.064	1.27	-0.101	-0.147	-0.009	-0.004	0.023	0.016
2	eFlanker	ΔACC (T:neg/D:all) - (T:neu/D:all)	-0.282	0.159	1.857	-0.209	-0.182	-0.13	-0.125	-0.21	-0.198
2	eFlanker	ΔACC (T:neg/D:all) - (T:pos/D:all)	-0.125	-0.037	3.807	-0.128	-0.17	-0.064	-0.074	-0.077	-0.084
2	eFlanker	ΔACC (T:emo/D:cong) - (T:neu/D:neu)	-0.225	0.225	0.372	**-0.289**	-0.235	-0.07	-0.065	-0.149	-0.123
2	eGoNoGo	ΔACC T:neg – T:pos	0.043	-0.154	0.199	0.148	0.087	0.115	0.083	0.206	0.197
2	eStroop	ΔRT (T:emo/D:all) – (T:neu/D:all)	0.032	-0.071	0.723	0.069	0.05	0.015	0.002	-0.096	-0.107
2	eStroop	ΔRT (T:pos/D:all) – (T:neu/D:all)	-0.103	0.13	0.098	-0.191	-0.167	-0.169	-0.156	0.09	0.11
2	eStroop	ΔRT (T:neg/D:all) – (T:neu/D:all)	0.116	-0.095	3.011	-0.001	-0.026	0.085	0.07	-0.188	-0.206
3	eFlanker	ΔRT (T:neg/D:all) – (T:pos/D:all)	0.039	-0.072	1.587	0.051	0.03	-0.076	-0.078	-0.107	-0.114
3	eFlanker	ACC overall	0.285	**0.499**	0.042	-0.126	-0.013	-0.154	-0.167	**-0.279**	**-0.255**
3	eFlanker	ACC T: pos / D: neg	0.272	**0.317**	0.006	-0.053	0.052	0.029	0.043	-0.22	-0.197
3	eFlanker	ACC T: neg / D: pos	0.135	**0.307**	**4.955**	-0.02	0.053	0.033	0.046	-0.155	-0.133
3	eFlanker	ACC T: emo / D: incong	0.273	**0.39**	0.19	0.022	0.143	-0.031	-0.014	**-0.342**	**-0.329**
3	eFlanker	ACC T: pos / D: incong	0.274	**0.344**	0.022	-0.178	-0.06	-0.026	-0.003	**-0.406**	**-0.383**

3	eFlanker	ACC T: neg / D: incong	0.224	**0.381**	2.162	-0.162	-0.096	-0.204	-0.218	**-0.314**	**-0.29**
3	eFlanker	ΔACC (T:pos/D:incong) – (T:pos/D:cong)	0.141	0.112	0.619	0.118	0.156	0.093	0.091	-0.028	-0.018
3	eFlanker	ΔACC (T:neg/D:incong) – (T:neg/D:cong)	0.062	0.009	0.066	0.106	0.129	0.216	0.22	**-0.244**	**-0.249**
3	eFlanker	ΔACC (T:emo/D:incong) – (T:emo/D:cong)	0.146	0.204	0.033	0.21	**0.257**	0.215	0.218	-0.155	-0.142
3	eFlanker	ACC T: all / D: cong	0.222	**0.352**	0.01	-0.142	-0.07	-0.183	-0.197	-0.128	-0.1
3	eFlanker	ACC T: all / D: incong	0.297	**0.5**	0.146	-0.125	0.034	-0.118	-0.108	**-0.348**	**-0.333**
3	eFlanker	ΔACC (T:all/D:incong) – (T:all/D:cong)	0.109	0.17	0.234	0.137	0.164	0.142	0.142	-0.147	-0.135
3	eFlanker	ACC T: pos	0.262	**0.393**	1.065	-0.159	-0.067	0.02	0.005	-0.221	-0.186
3	eFlanker	ACC T: neu	0.207	**0.418**	0.032	-0.197	-0.085	-0.21	-0.206	-0.125	-0.096
3	eFlanker	ACC T: neg	0.232	**0.42**	**4.496**	-0.208	-0.135	**-0.297**	**-0.315**	**-0.261**	-0.242
3	eFlanker	ACC T: emo	0.287	**0.432**	0.319	-0.162	-0.071	-0.114	-0.125	**-0.339**	**-0.322**
3	eFlanker	ΔACC T: pos – T: neu	0.13	0.045	2.344	**-0.26**	-0.238	-0.012	-0.008	-0.173	-0.165
3	eGoNoGo	ΔRT T:neg – T:pos	0.083	0.011	0.046	-0.038	-0.054	-0.048	-0.057	-0.136	-0.136
3	eGoNoGo	ACC overall	0.188	**0.333**	0.342	-0.17	-0.078	-0.018	0.036	**-0.311**	**-0.299**
3	eGoNoGo	ACC T: pos	0.139	**0.423**	0.017	-0.218	-0.113	-0.077	-0.018	**-0.292**	**-0.292**
3	eGoNoGo	ACC T: neg	0.207	0.195	3.383	-0.16	-0.116	-0.084	-0.056	-0.015	-0.002
3	eStroop	ΔRT T: neg – T: pos	0.027	-0.045	0.19	-0.1	-0.108	0.198	0.196	-0.237	-0.25
3	eStroop	ACC overall	0.165	**0.292**	0.326	-0.036	0.072	0.036	0.091	-0.169	-0.145
3	eStroop	ACC T:emo	0.173	0.216	2.672	-0.18	-0.105	-0.157	-0.111	-0.001	0.02
3	eStroop	ΔACC T:emo – T:neu	0.064	0.116	0.366	-0.144	-0.11	0.009	0.032	0.007	0.03

RT: reaction time, **ACC**: accuracy
Δ: difference between two conditions
T: target, **D**: distractor
pos: positive, **neg**: negative, **neu**: neutral, **emo**: emotional (pos or neg)
cong: congruent (i.e. target = distractor), **incong**: incongruent (i.e. target ≠ distractor)
#: “Age out” indicates that the age was covaried out during the calculation of the Pearson correlation coefficient.
The numbers in the bold font indicate a statistical significance with the P value under 0.05.

Table S2. We conducted a post-hoc analysis to examine whether any bias existed between the two methods of collecting the mental state scales (self-completed vs. experimenter-guided). The CES-DC scores obtained from the two methods were separately z-scored for standardization and then combined. Pearson correlation coefficients were then calculated between the standardized CES-DC scores and the E-scores while controlling for age. The same procedure was applied to the STAI-CH scale.

The association patterns between the E-scores and mental state scales were identical to the original findings, suggesting that any bias introduced by the two collection methods was negligible (self-completed vs. experimenter-guided).

All age-covaried out	**E-score 1**	**E-score 2**	**E-score 3**
**Depression**<br>**CES-DC** (standardized)	r=0.282 (P=.03)	r=0.515 (P<.001)	r=-0.094 (P=.46)
**Anxiety**<br>**STAI-CH** (standardized)	r=0.169 (P=.19)	r=0.34 (P=.006)	r=-0.143 (P=.26)

Table S3. To further disentangle the effects of depression, anxiety, and ADHD on task performance (i.e., E-scores), we conducted partial correlation analyses between each E-score and the CES-DC, STAI-Ch, or K-ARS while controlling for the remaining scales and age. First, the associations between E-score 1 and 2 and depression (CES-DC) remained significant even after controlling for anxiety, ADHD, and age (E-score 1: r=0.286, P=.03; Escore 2: r=0.4, P=.003). Similarly, the partial correlation between E-score 3 and ADHD remained significant when controlling for anxiety, depression, and age (r=−0.324, P=.02). In contrast, the previously observed correlation between anxiety (STAI-Ch) and E-score 2 was no longer significant when controlling for depression (r=0.061, P=.65).

We further clarified these findings using a multiple regression analysis, which yielded a consistent pattern. In the model "E-score 2 (Y) = STAI-Ch (X_1) + CES-DC (X_2) + K-ARS (X_3) + age (X_4)", anxiety (STAI-Ch) no longer predicted E-score 2 (t=−0.154, P=.88), whereas depression (CES-DC) remained a significant predictor (t=3.175, P=.002).

Together, these results suggest that the relationship between E-score 2 and anxiety (STAI-Ch) does not reflect an anxiety-specific effect; rather, it appears to arise from overlapping characteristics shared between the anxiety and depression measures.

Partial correlation	**E-score 1**	**E-score 2**	**E-score 3**
**Depression: CES-DC**	r=0.286	r=0.4	r=-0.013
(controlling for STAI-Ch, K-ARS, and age)	P=.03	P=.003	P=.93
**Anxiety: STAI-Ch**	r=0.01	r=0.061	r=-0.104
(controlling for CES-DC, K-ARS, and age)	P=.94	P=.66	P=.45
**ADHD: K-ARS**	r=-0.113	r=0.095	r=-0.324
(controlling for CES-DC, STAI-Ch, and age)	P=.41	P=.49	P=.02

Supplementary Figure 1. Selection of principal components for calculating E-scores

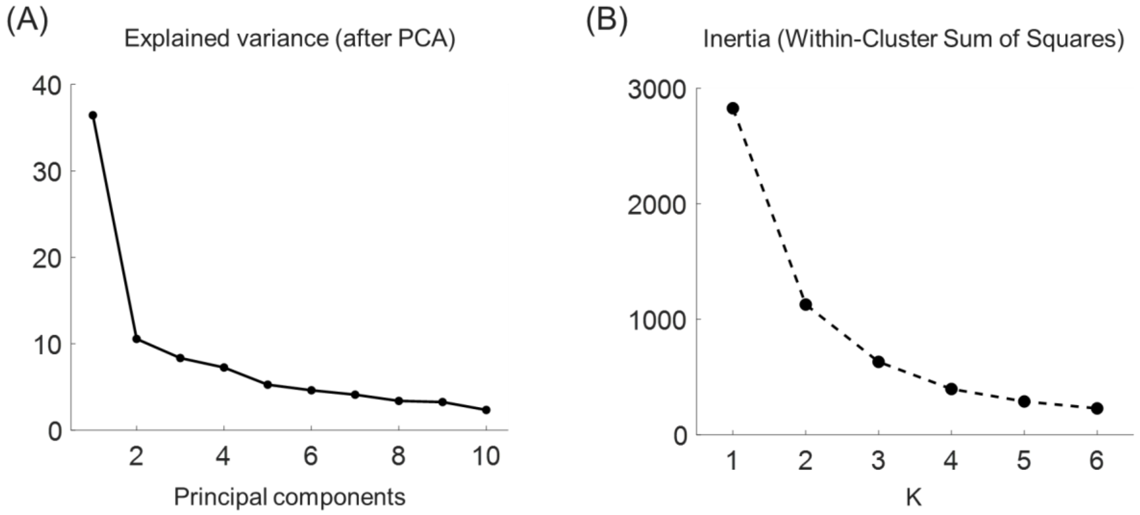

E-scores were calculated to summarize a range of behavioral indices in the children's performance on the emotion-cognitive tasks. First, 82 behavioral indices were reduced into two-dimensional latent features of task performance were calculated by extracting the first and second principal components of the z-scored behavioral indices. **(A)** The selection of two principal components for K-means clustering was based on the amount of variance explained; only the first two principal components accounted for more than 10% of the total variance. Then, the K-means clustering method was applied to the two principal components for dividing the latent features into several groups based on their squared Euclidean distance. **(B)** We validated our selection of three clusters for feature grouping by calculating inertia (within-cluster sum of squared error). Inertia measures the compactness of the clusters by calculating the sum of the squared distances between each data point and its assigned centroid. Lower inertia generally indicates better clusters (more robust), but it naturally decreases as K increases. Therefore, similar to other prior studies, the elbow method was used to find a point where the rate of decrease in inertia slows down, suggesting an optimal K as 3.
